# The use of thyroid hormones in the treatment of euthyroid patients with treatment-resistant depression. Data from a survey of 5695 European national endocrine professional organization members

**DOI:** 10.3389/fendo.2025.1665720

**Published:** 2025-09-23

**Authors:** Stephen Ludgate, Anne McGowan, Carla Moran, Roberto Attanasio, Miloš Žarković, Endre Vezekenyi Nagy, Roberto Negro, Enrico Papini, Chagit Adler Cohen, Ersin Akarsu, Maria Alevizaki, Göksun Ayvaz, Tomasz Bednarczuk, Biljana Nedeljković Beleslin, Eszter Berta, Miklos Bodor, Anna Maria Borissova, Mihail Boyanov, Camille Buffet, Maria-Cristina Burlacu, Jamina Ćirić, Juan J. Díez, Harald Dobnig, Valentin Fadeyev, Benjamin C. T. Field, Eric Fliers, Dagmar Führer-Sakel, Juan Carlos Galofré, Tommi Hakala, Jan Jiskra, Peter A. Kopp, Michael Krebs, Michal Kršek, Martin Kužma, Ivica Lazúrová, Laurence Leenhardt, Vitaliy Luchytskiy, Miguel Melo, Saara Metso, Tatyana Morgunova, Dan Alexandru Niculescu, Božidar Perić, Tereza Planck, Catalina Poiana, Francisca Marques Puga, Eyal Robenshtok, Patrick Rosselet, Marek Ruchala, Kamilla Ryom Riis, Alla Shepelkevich, Mykola D. Tronko, Jacob Stampe Frølich, David Unuane, Irfan Vardarli, W. Edward Visser, Andromachi Vryonidou, Younes Ramazan Younes, Elena Yurenya, Petros Perros, Laszlo Hegedüs

**Affiliations:** ^1^ The School of Medicine, Trinity College Dublin, The University of Dublin, Dublin, Ireland; ^2^ Robert Graves Institute, Tallaght University Hospital, Dublin, Ireland; ^3^ Diabetes and Endocrinology Section, Beacon Hospital, Dublin, Ireland; ^4^ School of Medicine, University College Dublin, Dublin, Ireland; ^5^ Endocrine Department, St Vincent’s University Hospital, Dublin, Ireland; ^6^ Scientific Committee Associazione Medici Endocrinologi, Udine, Italy; ^7^ Faculty of Medicine, University of Belgrade, Belgrade, Serbia; ^8^ Division of Endocrinology, Department of Medicine, Faculty of Medicine, University of Debrecen, Debrecen, Hungary; ^9^ Division of Endocrinology V.Fazzi Hospital, Department of Experimental Medicine, University of Salento, Lecce, Italy; ^10^ Department of Endocrinology and Metabolism, Regina Apostolorum Hospital, Lifenet Health Group, Rome, Italy; ^11^ Rabin Medical Center, Tel-Aviv University, Tel-Aviv, Israel; ^12^ Department of Internal Medicine, Division of Endocrinology, Faculty of Medicine, Gaziantep University, Gaziantep, Türkiye; ^13^ Endocrine Unit and Diabetes Centre, Department of Clinical Therapeutics, Alexandra Hospital, School of Medicine, National and Kapodistrian University of Athens, Athens, Greece; ^14^ Koru Ankara Hospital, Department of Endocrinology and Metabolism, Ankara, Türkiye; ^15^ Department of Internal Medicine and Endocrinology, Medical University of Warsaw, Warsaw, Poland; ^16^ Clinic of Endocrinology and Metabolism, University Hospital “Sofiamed”, Medical Faculty, Sofia University “Saint Kliment Ohridski”, Sofia, Bulgaria; ^17^ Clinic of Endocrinology and Metabolism, University Hospital “Alexandrovska”, Sofia, Bulgaria; ^18^ Department of Internal Medicine, Medical University, Sofia, Bulgaria; ^19^ Sorbonne Universitè, GRC n16, GRC Thyroid Tumors, Thyroid Pathology and Endocrine Tumor Department, AP-HP, Hôpital Pitié-Salpêtriére, Paris, France; ^20^ Department of Endocrinology and Nutrition, Cliniques Universitaires St-Luc, Université Catholique de Louvain, Brussels, Belgium; ^21^ Department of Endocrinology, Hospital Universitario Puerta de Hierro Majadahonda, Madrid, Spain; ^22^ Instituto de Investigación Sanitaria Puerta de Hierro Segovia de Arana, Madrid, Spain; ^23^ Department of Medicine, Universidad Autónoma de Madrid, Madrid, Spain; ^24^ Thyroid Endocrinology Osteoporosis Institute Dobnig, Graz, Austria; ^25^ Thyroid Practice for Radiofrequency Ablation, Vienna, Austria; ^26^ Department of Endocrinology No. 1, N.V. Sklifosovsky Institute of Clinical Medicine, I.M. Sechenov First Moscow State Medical University, Moscow, Russia; ^27^ Section of Clinical Medicine, Faculty of Health and Medical Sciences, University of Surrey, Guildford, United Kingdom; ^28^ Department of Endocrinology & Metabolism, Amsterdam UMC, University of Amsterdam, Amsterdam, Netherlands; ^29^ Department of Endocrinology, Diabetes and Metabolism, University Hospital Essen, University-Duisburg-Essen, Essen, Germany; ^30^ Department of Endocrinology, Clínica Universidad de Navarra, Pamplona, Spain; ^31^ Instituto de Investigación Sanitaria de Navarra, Pamplona, Spain; ^32^ Department of Surgery, Tampere University Hospital, Tampere, Finland; ^33^ 3^rd^ Department of Medicine, 1^st^ Faculty of Medicine, Charles University, General University Hospital, Prague, Czechia; ^34^ Division of Endocrinology, Diabetology and Metabolism, University of Lausanne, Lausanne, Switzerland; ^35^ Internal Medicine III, Division of Endocrinology, Medical University of Vienna, Vienna, Austria; ^36^ 5^th^ Department of Internal Medicine, Medical Faculty of Comenius University and University Hospital, Bratislava, Slovakia; ^37^ P.J. Šafárik University Košice, 1^st^ Department of Internal Medicine of the Medical Faculty, Košice, Slovakia; ^38^ Department of Reproductive Endocrinology, Institute of Endocrinology and Metabolism Named After V.P. Komissarenko, National Academy of Medical Science of Ukraine, Kyiv, Ukraine; ^39^ Department of Endocrinology, Diabetes and Metabolism Coimbra Local Health Unit, Medical Faculty, University of Coimbra, Coimbra, Portugal; ^40^ Department of Endocrinology, Tampere University Hospital, Tampere, Finland; ^41^ Department of Endocrinology, Carol Davila University of Medicine and Pharmacy, Bucharest, Romania; ^42^ Department of Endocrinology, Diabetes and Metabolic Diseases “Mladen Sekso”, University Hospital Center “Sisters of Mercy”, Zagreb, Croatia; ^43^ Department of Endocrinology, Skåne University Hospital, Malmö, Sweden; ^44^ Serviço de Endocrinologia, Diabetes e Metabolismo, ULS São João, Porto, Portugal; ^45^ Endocrinology Institute, Rabin Medical Center, Gray Faculty of Medicine, Tel Aviv University, Tel-Aviv, Israel; ^46^ Cabinet Médical 2, Rue Bellefontaine, Lausanne, Switzerland; ^47^ Department of Endocrinology, Metabolism and Internal Medicine, Poznan University of Medical Sciences, Poznan, Poland; ^48^ Department of Endocrinology, Odense University Hospital, Odense, Denmark; ^49^ Department of Endocrinology, Belarusian State Medical University, Minsk, Belarus; ^50^ V.P. Komissarenko Institute of Endocrinology and Metabolism, National Academy of Medical Science of Ukraine, Kyiv, Ukraine; ^51^ Department of Internal Medicine, Endocrine Unit, UZ Brussel, Vrije Universiteit Brussel, Brussels, Belgium; ^52^ Department of Medicine I, Klinikum Vest GmbH Knappschaftskrankenhaus Recklinghausen, Academic Hospital, Ruhr-University Bochum, Recklinghausen, Germany; ^53^ 5^th^ Medical Department, Division of Endocrinology and Diabetes, Medical Faculty Mannheim, Heidelberg University, Mannheim, Germany; ^54^ Rotterdam Thyroid Center, Department of Internal Medicine, Erasmus MC, Rotterdam, Netherlands; ^55^ Department of Endocrinology and Diabetes Centre, Hellenic Red Cross Hospital, Athens, Greece; ^56^ East Surrey Hospital, Surrey and Sussex Healthcare NHS Trust, Redhill, United Kingdom; ^57^ Minsk Endocrinology Medical Center, Minsk, Belarus; ^58^ Translational and Clinical Research Institute, Newcastle University, Newcastle upon Tyne, United Kingdom

**Keywords:** TRD (treatment-resistant depression), thyroid, thyroid hormone, liothyroinine, levothyroxine

## Abstract

**Purpose:**

Treatment-resistant depression (TRD) is most commonly defined as depression that has not responded to two different pharmacological agents used for an adequate period of time. We explored the views of European specialists via survey, regarding the use of thyroid hormone (TH) in euthyroid patients with TRD as part of ‘Treatment of Hypothyroidism in Europe by Specialists: An International Survey’ (THESIS).

**Methods:**

The question “Thyroid hormones may be indicated in biochemically euthyroid patients with treatment resistant depression” was posed to specialists from 28 countries.

**Results:**

5695 valid responses were received following 17,232 invitations (33.0% response rate; 65% female, 90% endocrinologists). 348 (6.1%) stated that TH may be indicated in biochemically euthyroid patients with TRD. This view was more common in males (p<0.01), respondents who saw ≥100 patients with hypothyroidism per year (p<0.01), respondents who worked in private practice (p=0.05) and respondents who were not members of international specialist associations (p=0.05). Geographical variation existed with respondents in Eastern Europe significantly more likely to use TH in TRD (p<0.01). Linear regression showed a statistically significant reduction in the use of TH for TRD with increasing gross national income (F-statistic=7.35, CI -0.15 - -0.02, p=0.01). TH in TRD was recommended in psychiatry guidelines but not endocrinology guidelines.

**Conclusion:**

While there is limited evidence for their use, over 6% of respondents stated that TH may be indicated in TRD. Due to the risk of iatrogenic thyrotoxicosis and increased morbidity the use of TH should be addressed in relevant endocrinology guidelines and consensus should be reached between specialties.

## Introduction

Treatment of Hypothyroidism in Europe by Specialists: An International Survey (THESIS) is a large European survey examining the views of the use of thyroid hormones (TH) among thyroid specialists in Europe. The survey was undertaken between 2019–2021 and most countries have previously published their national data (1–21).

The purpose of the THESIS survey was to evaluate the clinical practices of thyroid specialists across Europe for both evidence-based purposes as well as non-typical uses for TH (22, 23). Here, we report data with respect to thyroid specialists’ views on the use of TH in biochemically euthyroid patients with treatment-resistant depression (TRD).

Inconsistency in the literature exists regarding the definition of TRD and a unified international guideline definition is lacking. The most frequently used definition is a diagnosis of depression that previously failed two treatments with antidepressant medications, following adequate dose and duration (24).

The association between the thyroid gland and psychiatric disorders was first described by Parry in his posthumous paper in 1825 where he described an increased incidence of “nervous affectations” in patients with thyroid disorders (25). Over the last 50 years, data examining the relationship between the thyroid gland and psychiatric disorders, specifically depression has emerged, and there is a bi-directional association between hypo- as well as hyperthyroidism and psychiatric disorders, including depression (26, 27). Changes in the hypothalamic-pituitary-thyroid (HPT) axis in patients experiencing depression have been investigated and subsequently the role of TH in treating depression on the HPT axis has been explored (28). The hypothesis for many of these studies is that changes observed in the HPT-axis may be explained by cerebral serotonin deficiency and treatment with liothyronine (T3) may reverse this change (28, 29).

Studies exist examining the connection between hypothyroidism and depression and a number of trials have been performed examining the role of TH in the treatment of depression (26, 27). Initial trials examining T3 as an augmentation agent for individuals who did not respond to antidepressants took place in the 1960s and 1970s (30–32). Those trials reported a benefit in supplementing standard treatment with T3 in patients with depression. A meta-analysis published in 2001 supported the use of T3 in the augmentation of tricyclic antidepressants (33). Since then, a number of placebo-controlled trials examining the role of T3 in the treatment of depression and TRD were performed. A large systematic review and network meta-analysis in 2022 examining augmentation agents in TRD suggested that T3 was an effective augmentation agent in the disease (34). However, a Danish systematic review and meta-analysis in 2020 did not support the use of TH in TRD and a 2015 review of augmentation agents also suggested that additional data were required to advise the use of TH in TRD, so its use remains controversial (35, 36). Despite this, TH is currently recommended as an augmentation agent in TRD in a number of national and international psychiatric guidelines (37, 38). Its use, however, is not currently recommended in any national or international guidelines on hypothyroidism (39). There is little evidence to support the use of levothyroxine (T4) in the management of TRD and it is not currently recommended in any national or international guideline on depression or hypothyroidism (37, 39).

The aim of this manuscript was to document the characteristics and views of thyroid specialists from the 28 countries who participated in the THESIS survey with respect to the use of TH in biochemically euthyroid patients with TRD.

## Material and methods

Ethical approval for this study was obtained from the relevant institutional review board, and the research was conducted in accordance with the principles outlined in the Declaration of Helsinki. This study is a sub-analysis of the THESIS survey, which has previously been described in detail (22, 23). It is an anonymous online survey which was conducted between 2019 and 2021. The online questionnaire (see **Supplementary Data**) consisted of eight questions about physician characteristics and twenty-three questions concerning the use of TH in various clinical settings. The questionnaire was initially developed in English and then tested in a pilot study which was distributed to Italian endocrinologists (1).

Guidelines for internet-based electronic surveys (CHERRIES) were followed in this study (40). European countries with a population of over 4 million people were selected for the survey and thyroid specialists who were members of national endocrine and/or thyroid scientific professional organizations were targeted. The project was overseen by a Steering Committee (LH, EVN, EP, PP, RA, and RN). Two national leads from each participating country, along with the Steering Committee, ensured the authenticity of the data collected.

The key question for this analysis was whether “Thyroid hormones may be indicated in biochemically euthyroid patients with depression resistant to antidepressant medications.” Previous sub analyses examined responses for the use of TH in antibody positive women with unexplained infertility, euthyroid patients with enlarging goiter and euthyroid patients with obesity (41–43).

Geographic regions were defined in accordance with the United Nations Statistics Division (UNSD) definition: Northern Europe (Denmark, Finland, Ireland, Sweden, UK); Western Europe (Austria, Belgium, France, Germany, the Netherlands, Switzerland); Southern Europe (Croatia, Greece, Italy, Portugal, Serbia, Slovenia, Spain); Eastern Europe (Belarus, Bulgaria, Czech Republic, Hungary, Poland, Romania, Russian Federation, Slovakia, Ukraine); and Western Asia (Israel, Turkey) (44). Data on gross national income (GNI) per capita in USD via the Atlas methodology was taken from the World Bank Data Catalogue from 2024 (45). Rates of antidepressant prescribing in Europe were taken from the Organisation of Economic Co-operation and Development (OECD) pharmaceutical consumption data on antidepressants for the year 2020, where data was available for countries that participated in the THESIS survey (46). These data is given in defined daily doses per 1000 inhabitants per day.

On completion of the survey, authors established if each country had (A) national guidelines for the treatment of thyroid diseases, including hypothyroidism, (B) national guidelines for the treatment of depression or specific guidelines for TRD and (C) whether these guidelines addressed the use of TH in depression or TRD. In cases where national guidelines were absent, project leaders investigated whether any specific international guidelines were officially recommended by each country’s national society, and whether these guidelines supported or discouraged the use of TH in depression or TRD. Where it was unclear whether national guidelines existed, national leads were contacted.

### Statistical analyses

Only responses containing complete demographic information were included in the analysis. Statistical analyses were conducted using ‘R’ software (47). Survey data were not weighted, reflecting the characteristics of the information collected. Qualitative variables were presented as frequencies or proportions, while quantitative variables were presented as means with standard deviations.

To evaluate associations between qualitative variables, chi-square and Cramer’s V tests were employed. When applicable, linear regression analyses were performed using the statistical and ordinal R packages (48). A significance level of 5% was applied to all analyses. Effect size, reported alongside p-values, was used to determine the practical relevance of results, following guidelines to avoid emphasizing statistically significant but practically irrelevant findings (49, 50). Cramer’s V was interpreted using Rea and Parker’s scale, where values below 0.1 indicated insignificant associations, values between 0.1 and 0.2 indicated weak associations, values between 0.2 and 0.4 were moderate, values between 0.4 and 0.6 were relatively strong, and values above 0.6 indicated strong associations (49).

## Results

### Baseline characteristics of all respondents

We received 5695 valid responses following 17,232 invitations (33.0% valid response rate). The characteristics of these respondents have previously been described in detail (22, 23). The characteristics are summarized in **Table 1** and the response rates of each group as to whether they prescribe TH in biochemically euthyroid patients with TRD. Of note, 65.0% were female (3700/5695), 90.1% were endocrinologists (5132/5695) and the mean age was 49 ± 12 years. 78.8% of respondents had more than 10 years in clinical practice (4487/5693) and 83.4% treated over 50 patients with hypothyroidism each year (4732/5677).

**Table 1:** Table showing number of respondents in each category, response of each group, p value and Cramers’ V for each characteristic.

Respondent Characteristics	Total N = 5695	TH Prescribers N = 348	TH Non-Prescribers N = 5347	P value	Cramer’s V
Sex				**<0.01**	0.035
Female	3700	203 (5.5)	3497 (94.5)		
Male	1995	145 (7.3)	1850 (92.7)		
Age (yrs)				0.52	0.027
≤30	282	19 (6.7)	263 (93.3)		
31-40	1375	72 (5.2)	1303 (94.8)		
41-50	1565	90 (5.8)	1475 (94.2)		
51-60	1479	101 (6.8)	1378 (93.2)		
61-70	792	52 (6.6)	740 (93.4)		
≥70	202	14 (6.9)	188 (93.1)		
Speciality				0.06	0.025
Endocrinologists	5132	303 (5.9)	4829 (94.1)		
Non-endocrinologists	563	45 (8)	518 (92)		
Volume in hypothyroidism treatment (patients/year)				**<0.01 (< 100 vs ≥100 patients/year)**	0.044
Rarely	158	10 (6.3)	148 (93.7)		
10 - 50	787	34 (4.3)	753 (95.7)		
50 - 100	1206	58 (4.8)	1148 (95.2)		
≥100	3526	246 (7)	3280 (93)		
Missing Data	18				
Years in professional practice				0.33	0.028
≤10	1206	64 (5.3)	1142 (94.7)		
11-20	1601	90 (5.6)	1511 (94.4)		
21-30	1476	100 (6.8)	1376 (93.2)		
31-40	1010	64 (6.3)	946 (93.7)		
≥40	400	30 (7.5)	370 (92.5)		
Missing data	2				
Private Practice				**0.05**	0.027
No	4097	234 (5.7)	3863 (94.3)		
Yes	1598	114 (7.1)	1484 (92.9)		
Academic Practice				0.84	0.003
No	3514	217 (6.2)	3297 (93.8)		
Yes	2181	131 (6)	2050 (94)		
Member of Society (International; ATA, ETA, LATS, AOTA/ National Society)				**0.05**	0.027
No	754	61 (8.1)	693 (91.9)		
Yes	4413	270 (6.1)	4143 (93.9)		
Missing Data	528 (France)				

Bold = statistically significant.

### Baseline characteristics and demographics of respondents who consider TH for biochemically euthyroid patients with TRD

Of the valid responses received, 348 (6.1%) of the 5695 respondents stated that TH might be indicated in the treatment of biochemically euthyroid patients with TRD (**Table 1**). Male respondents more commonly thought that TH was indicated compared to female respondents (7.3% *vs*. 5.5% p<0.01, Cramer’s V 0.035). Physicians who were not members of international endocrinology organizations or societies were more likely to prescribe TH in TRD (8.1% *vs*. 6.1%, p = 0.05, Cramer’s V = 0.027).

Of those respondents who considered TH in biochemically euthyroid patients with TRD, 70.7% (246/348) treated ≥100 patients with hypothyroidism per year (7% *vs* 4.7%, p < 0.01, Cramer’s V = 0.044). Of these 246 respondents, 94.7% were endocrinologists (233/246) with 36.2% (89/246) practicing in an academic center. In the THESIS survey, non-endocrinologists more commonly considered prescribing TH in biochemically euthyroid patients with TRD than endocrinologists, however this was not statistically significant (8% *vs*. 5.9%, p = 0.06, Cramer’s V = 0.025).

Age of respondents or years in clinical practice were not associated with differences in the rates of prescribing TH in biochemically euthyroid patients with TRD.

Academic practice was also not associated with differences in TH prescribing in TRD, however physicians engaging in private practice were more likely to use TH for this indication (7.1% *vs*. 5.7%, p = 0.05, Cramer’s V = 0.027).

### National and regional variations associated with prescribing of TH in biochemically euthyroid patients with TRD

**Figure 1:**
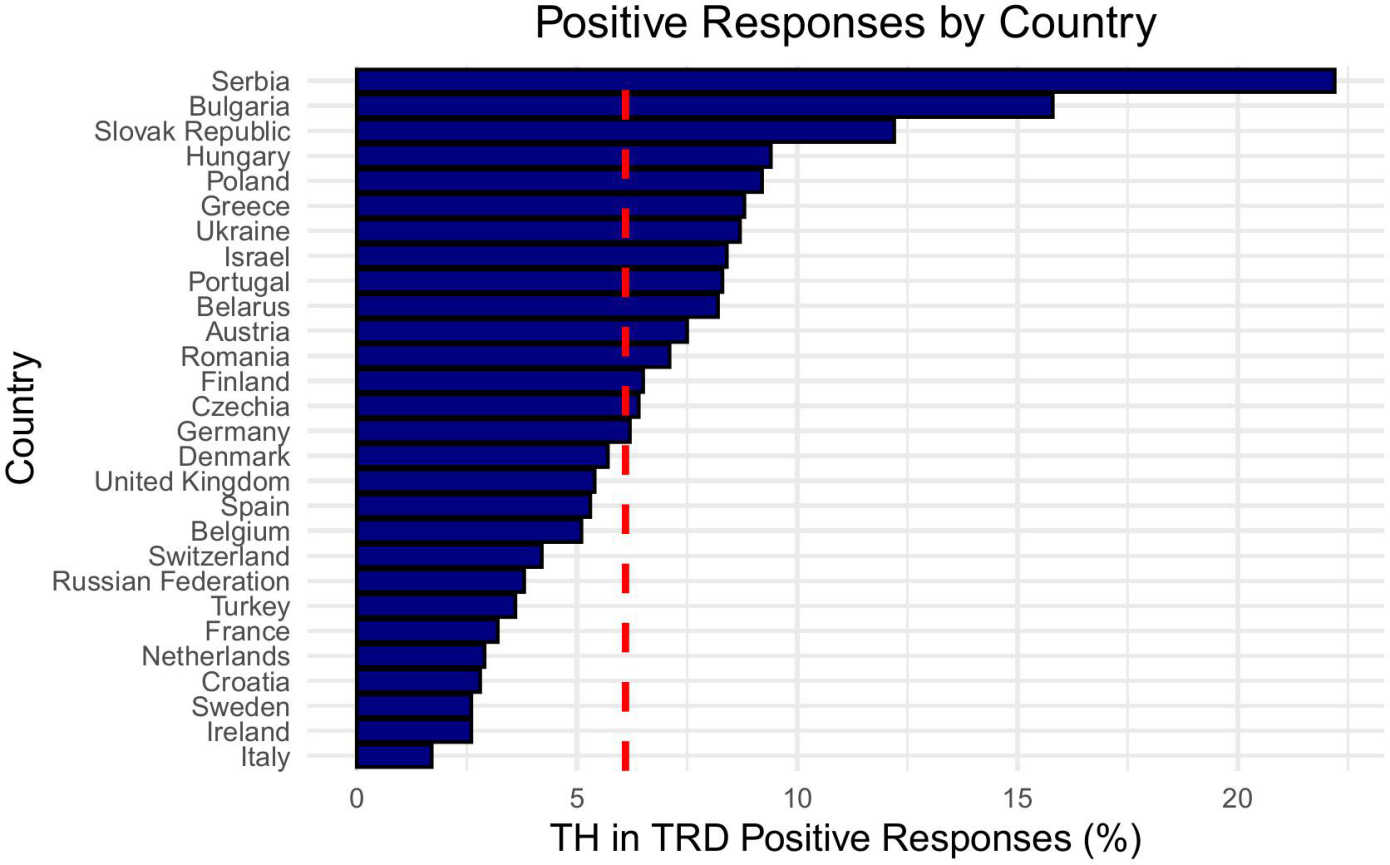
Bar chart showing positive responses by country, ranked from highest to lowest. Serbia leads with the highest percentage, followed by Bulgaria and the Slovak Republic. Italy has the lowest percentage. The x-axis represents percentage of positive responses, with a red dashed line showing the mean positive response rate of 6.1%.

**Figure 2:**
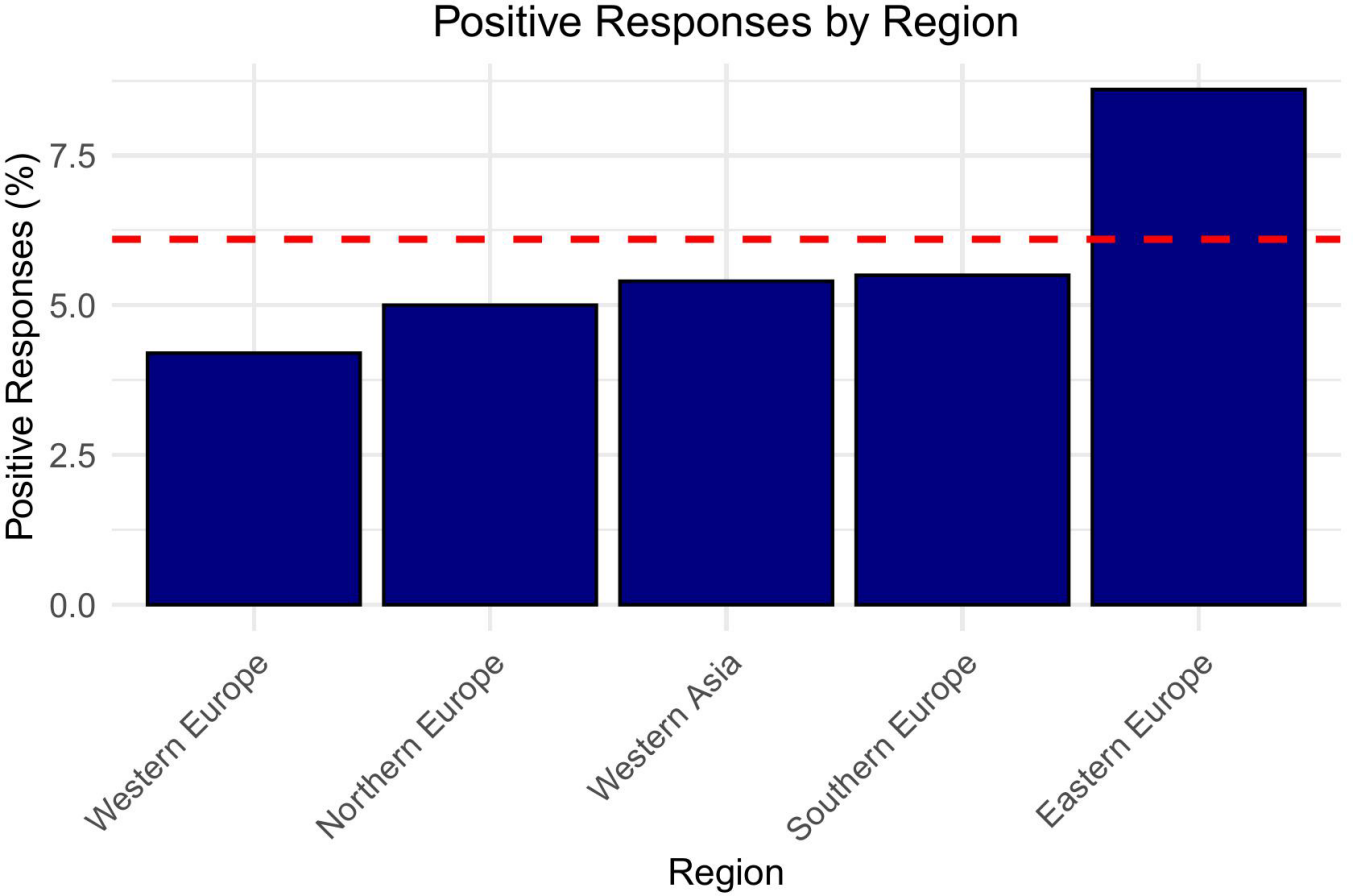
Bar graph titled “Positive Responses by Region” shows positive response percentages for five regions: Western Europe, Northern Europe, Western Asia, Southern Europe, and Eastern Europe. Eastern Europe has the highest percentage, above a red dashed line representing the mean positive response rate of 6.1%.

The responses from national regional groups showed a wide variation on the attitude towards the use of TH in biochemically euthyroid patients with TRD. These findings have been summarized in **Table 2**. The highest proposed rates of prescription of TH for TRD were seen in Serbia (22.2%), Bulgaria (15.8%) and Slovakia (12.2%) with the lowest prescription rates seen in Ireland, Sweden (both 2.6%) and Italy (1.7%) (**Figure 1**). There was a statistically significant difference between Eastern and Northern, Southern and Western European respondents combined with regards to the use of TH in TRD (8.6% *vs*. 5%, p < 0.01, Cramer’s V = 0.067) (**Figure 2**).

**Table 2:** Table showing the number of respondents with positive response rate (and %) for each country in the THESIS survey.

Country	Respondents (N)	N (%)
Western Europe	**938**	**39 (4.2)**
Austria	40	3 (7.5)
Belgium	79	4 (5.1)
France	528	17 (3.2)
Germany	161	10 (6.2)
Netherlands	35	1 (2.9)
Switzerland	95	4 (4.2)
Northern Europe	**713**	**36 (5)**
Denmark	158	9 (5.7)
Finland	123	8 (6.5)
Sweden	116	3 (2.6)
Ireland	39	1 (2.6)
United Kingdom	277	15 (5.4)
Southern Europe	**2053**	**112 (5.5)**
Croatia	71	2 (2.8)
Greece	441	39 (8.8)
Italy	843	14 (1.7)
Portugal	109	9 (8.3)
Serbia	99	22 (22.2)
Spain	490	26 (5.3)
Eastern Europe	**1679**	**144 (8.6)**
Belarus	146	12 (8.2)
Bulgaria	120	19 (15.8)
Czechia	157	10 (6.4)
Hungary	160	15 (9.4)
Poland	425	39 (9.2)
Romania	296	21 (7.1)
Russian Federation	131	5 (3.8)
Slovak Republic	49	6 (12.2)
Ukraine	195	17 (8.7)
Western Asia	**312**	**17 (5.4)**
Israel	119	10 (8.4)
Turkey	193	7 (3.6)

Bold = Values for regions.

### Prevalence of prescription of anti-depressant medications

**Figure 3:**
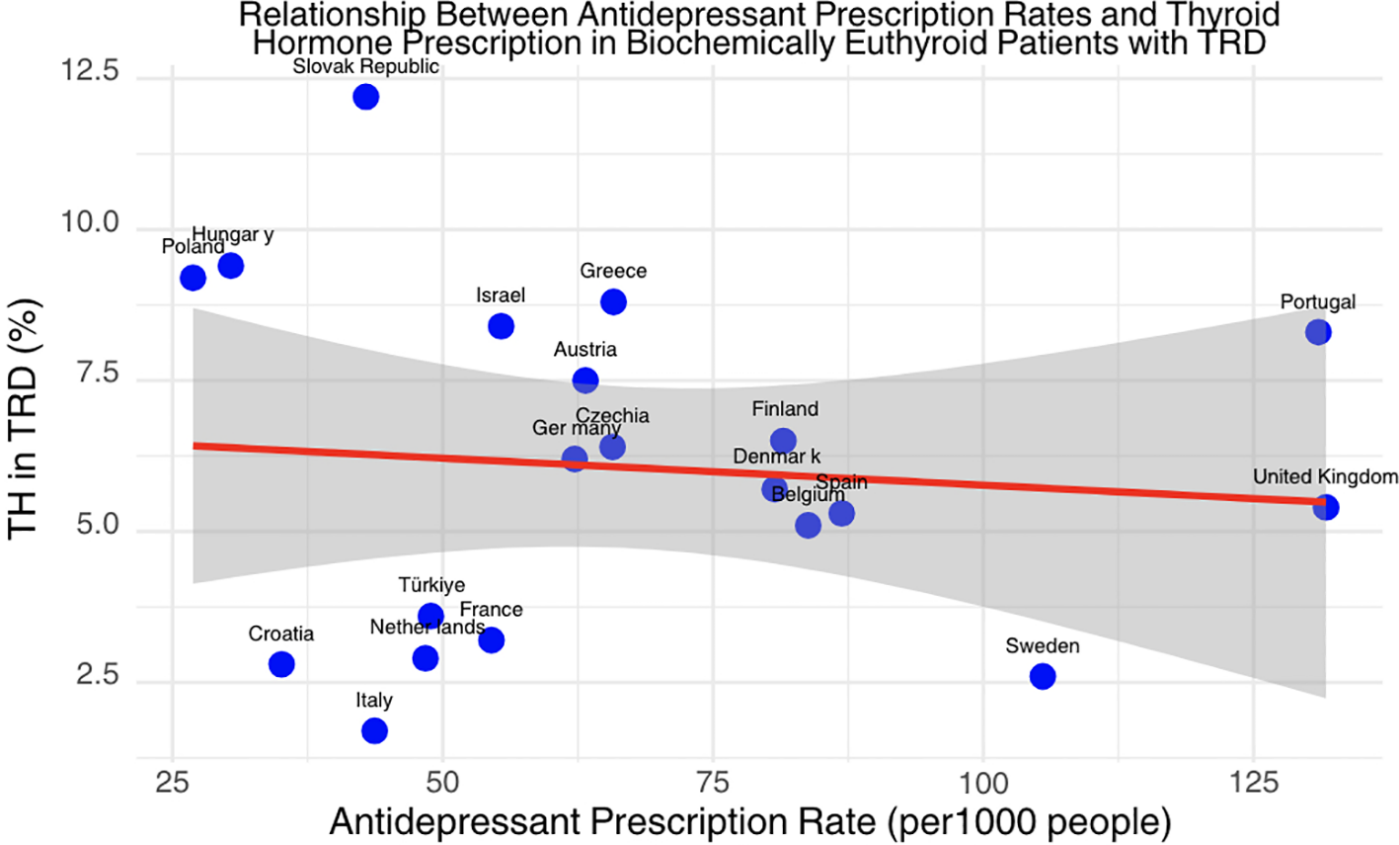
Scatter plot showing the relationship between antidepressant prescription rates and thyroid hormone prescription rates in biochemically euthyroid patients with TRD. The x-axis represents antidepressant prescription rates per 1,000 people, and the y-axis represents the percentage of thyroid hormone use in TRD.

Countries included in the THESIS study, which were also included in the OECD antidepressant consumption data in 2020, demonstrate heterogeneity in prescribing anti-depressant medication across the participating nations. Anti-depressant medications were most commonly prescribed in the UK, Portugal and Sweden and least commonly in Croatia, Hungary and Poland. We found that TH use in TRD was not statistically associated with the rates of antidepressant prescribing in countries with available data (F- statistic: 0.1661, CI: −0.055 to 0.037, p-value: 0.6884) (**Figure 3**).

### Gross national income and use of TH in TRD

**Figure 4:**
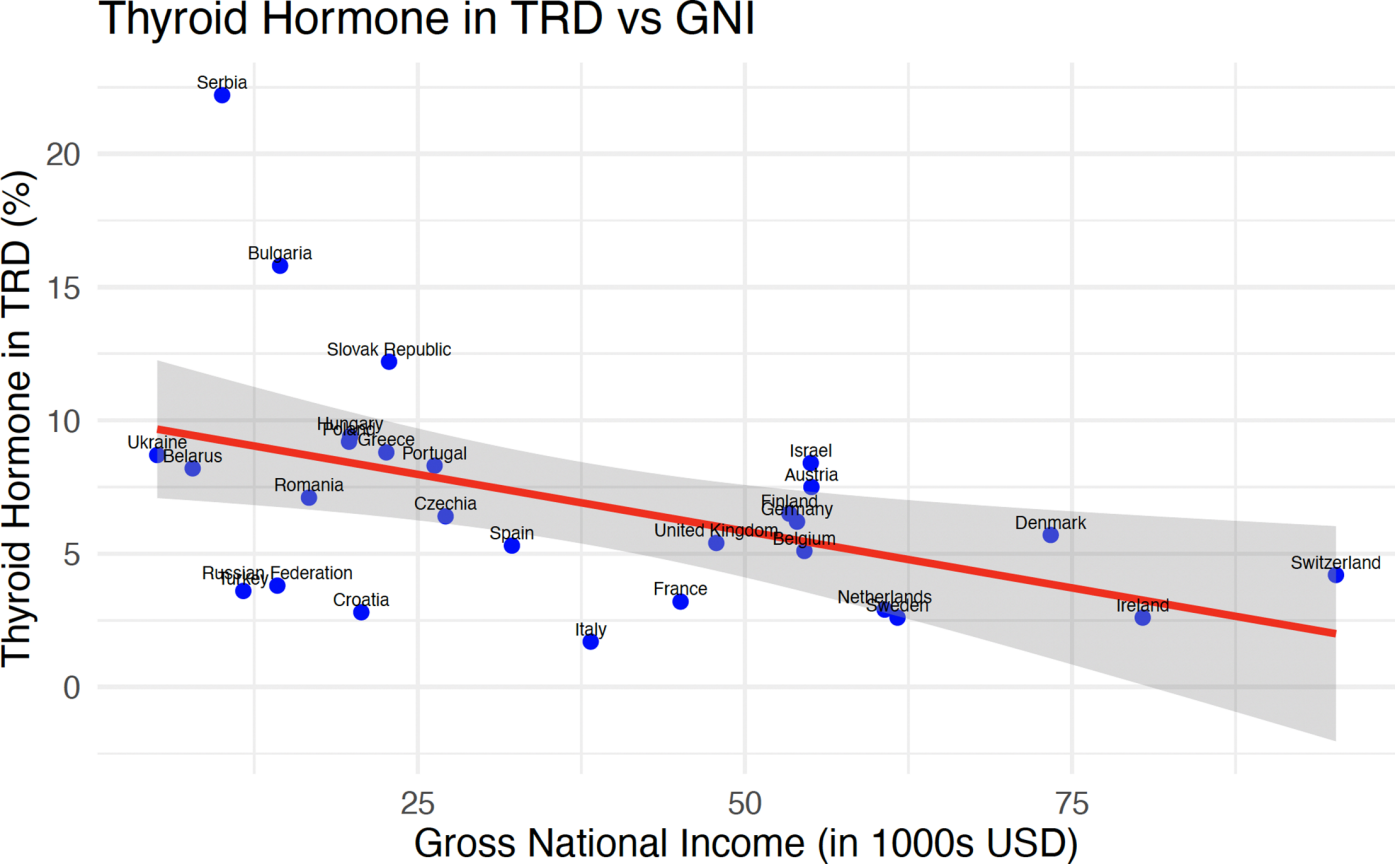
Scatter plot showing the relationship between thyroid hormone levels in TRD and GNI in thousands of USD.

We calculated linear regression of rates of TH use in TRD in countries *vs* GNI. This showed a statistically significant reduction in the use of TH for TRD with increasing GNI (F statistic = 7.35, CI -0.15 - -0.02, p = 0.01) (**Figure 4**).

### Guidelines and use of TH in TRD

Of the 28 countries that participated in the THESIS survey, 13 had national guidelines for hypothyroidism. Of these 13 countries, no guidelines address TRD and only one guideline addresses the use of TH in euthyroid patients with depression, which does not recommend the use of TH in euthyroid patients with depression (51).

Six of the 28 countries who participated in the THESIS survey have specific guidelines for TRD, four of which (66%) recommend T3 in euthyroid patients with TRD, as an augmentation agent with antidepressant medications. A further 10 countries in the survey have national guidelines for depression. Two of these (20%) guidelines on depression recommend T3 in euthyroid patients with depression, again as an augmentation agent. A summary of all national guidelines is included in **Table 3**.

**Table 3:** Table documenting which countries have national guidelines on hypothyroidism and depression, and if they referenced TRD or levothyroxine use in depression/TRD.

Country	Thyroid Guidelines (Y/N)	TRD/Depression Mentioned	Treatment Recommended	TRD/Dep Guidelines	TH Mentioned	Treatment Recommended
Western Europe						
Austria	No	N/A	N/A	Yes (TRD)	Yes	Y (WFSBP)
Belgium	Yes	No	N/A	No	No	No
France	Yes	No	N/A	Yes (TRD)	Yes	Y (Add on)
Germany	No	N/A	N/A	Yes	No	No
Netherlands	Yes	No	N/A	Yes	No	No
Switzerland	No	N/A	N/A	Yes	No	No
Northern Europe						
Denmark	Yes	No	N/A	Yes	No	No
Finland	No	N/A	N/A	Yes	No	No
Sweden	No	N/A	N/A	Yes	No	No
Ireland	No	N/A	N/A	No	N/A	N/A
United Kingdom	Yes	No	N/A	Yes (TRD)	Yes	T3 as add-on (off label)
Southern Europe						
Croatia	No	N/A	N/A	No	No	No
Greece	No	N/A	N/A	No	N/A	N/A
Italy	Yes	Yes	Against LT4, More info needed LT3	Yes (TRD)	Yes	No
Portugal	Yes	No	N/A	Yes (TRD)	No	No
Serbia	Yes	No	N/A	Y	No	N/A
Spain	No	N/A	N/A	Yes	Yes	Yes (Add-on, care with SE’s)
Eastern Europe						
Belarus	N/A	N/A	N/A	N/A	N/A	N/A
Bulgaria	Yes	No	N/A	No	N/A	N/A
Czechia	Yes	No	N/A	No	N/A	N/A
Hungary	No	No	No	Yes	Yes	Yes (Add-on, care with SE’s)
Poland	No	N/A	N/A	Yes (TRD)	Yes	Yes (Add on however care with SE’s)
Romania	No	N/A	N/A	No	N/A	N/A
Russian Federation	Yes	No	N/A	Yes	No	No
Slovak Republic	No	No	No	No	N/A	N/A
Ukraine	N/A	N/A	N/A	N/A	N/A	N/A
West Asia						
Israel	No	N/A	N/A	No	N/A	N/A
Turkey	Yes	No	No	Yes	Yes	Yes

Table documenting which countries have national guidelines on hypothyroidism and depression, and if they referenced TRD.

A number of international guidelines are used by nations in the THESIS survey. The ATA guidelines discuss the use of both T4 and T3 in euthyroid patients with depression recommend neither (39). The European Thyroid Association (ETA) did not have a general guideline on hypothyroidism at the time of the THESIS survey and so did not address the use of TH in depression or TRD.

We reviewed six prominent international psychiatry guidelines which have previously been described as the ‘major American and European guidelines.’ (52) The World Federation of Societies of Biological Psychiatry (WFSBP), the American Psychiatric Association (APA), the British Association for Psychopharmacology (BAP), The Canadian Network for Mood and Anxiety Treatments (CANMAT), The Texas Medication Algorithm Project (TMAP) and the National Institute for Health and Care Excellence (NICE) guidelines on major depressive disorders and TRD, all recommend T3 as an adjunct treatment in euthyroid patients with TRD (37, 53–57).

## Discussion

The THESIS survey demonstrated that just over 6% of physicians treating hypothyroidism across Europe consider TRD as a potential indication for TH in biochemically euthyroid patients. While this figure is small, it still represents a significant number (348) of specialists surveyed. We noted that male specialists were more likely to prescribe TH in TRD than their female counterparts. It has previously been shown that female physicians are more likely to practice conservative prescribing and are also more likely to follow best practice guidelines (58–60). It is also documented that risk taking of all types is more common in males compared to females and a number of guidelines warn of potential side effects using TH in patients with TRD (53, 61). While the overall rate of use of TH in biochemically euthyroid patients with TRD in male specialists remains low (7.3%), these factors may account for differences observed.

Specialists who thought TH was indicated in biochemically euthyroid patients with TRD were more likely to see a higher number of patients with hypothyroidism (**Table 1**). It is possible that patients seeing these physicians had previously seen a number of other specialists and had exhausted alternative treatment options. In these instances, these physicians may be aware of the limited evidence for TH as an adjunct therapy in TRD (34). Some of the key trials showing evidence for TH in the treatment of TRD include a trial from 1993 which compared T3 as an adjuvant to standard treatment compared to placebo and lithium, with improvement observed in patients treated with T3 (62). A trial conducted in 1990 by the same group demonstrated that T3 was superior to T4 in the treatment of TRD (63). T3 has also been shown to accelerate the response to tricyclic antidepressants (33). The largest randomized controlled trial to date, carried out in 2011 by Fang et al, demonstrated a benefit to augmenting paroxetine with T3 with increasing remission rates in patients with TRD seen, as defined by the 17-item Hamilton Rating Scale for Depression, however this trial was limited by a small sample size (64). All of these trials are included in the 2022 systematic review and network meta-analysis that showed a statistically significant benefit to T3 as an augmentation agent in TRD (34). That publication included five studies that looked at T3 as an augmentation agent. This included head to head, placebo-controlled and mixed arm trials. Network meta-analyses compare direct, indirect and mixed evidence and it has previously been described how some conclusions from these analyses may not be clearly justified (65).

The use of TH for euthyroid patients with TRD remains controversial, however, and a number of studies have found no benefit in the treatment of TRD. A meta-analysis published in 2020 which examined papers investigating the use of TH in the treatment of TRD included 10 studies, with 663 patients, and concluded that there was not sufficient evidence for the use of TH in the treatment of unipolar TRD (35). One study included in this meta-analysis examined T3 and lithium as adjuvant therapies in the treatment of TRD and found that neither agent improved outcomes significantly (66). A placebo-controlled trial, carried out in 1987, did not show a benefit with the use of T3 in patients with depression who had failed imipramine treatment (67). A 2006 study by Joffe et al. also found no benefit in the use of T3 as an adjuvant treatment in a double blind, placebo controlled trial (68). A systematic review and meta-analysis published in 2023 on early augmentation agents in TRD noted large inter study differences between different agents as well as differing definitions of TRD which made interpretation difficult and warned against a one size fits all approach in patients with this condition (69). A systematic review and meta-analysis published in 2019 aimed to classify the effectiveness of augmentation therapies in TRD by examining randomized controlled trials with at least ten patients, however, only one study involving TH (Fang et al.) met the criteria for the analysis (70).

Specialists who recommended TH for euthyroid patients with TRD were less likely to be a member of an international society. This correlates with the absence of endocrinology guideline recommendation for the use of TH in TRD. Of the national guidelines on hypothyroidism, Italy’s guidelines were the only ones which addressed the use of TH in euthyroid patients with depression (51). They do not recommend the use of TH in euthyroid patients with depression. Austrian national guidelines on the treatment of TRD recommend T3 as an augmentation agent when monotherapy has failed and this guideline is adapted from the WFSBP guidelines (71). French guidelines on TRD recommend T3 as an augmentation agent to be used with a variety of antidepressants and the NICE guidelines in the UK advise T3 be used as an adjunct off-label with specialist input (38, 53). Polish guidelines reference four trials which showed a benefit of TH in TRD in their recommendation (72).

The WFSBP guidelines are internationally recognized for the treatment of major depressive disorders, including TRD (37). These guidelines recommend antidepressant augmentation with T3 where monotherapy has failed. The APA guidelines on major depressive disorders were last released in 2010 and recommended the use of TH as an augmentation agent with ‘moderate clinical confidence.’ (55) The BAP guidelines from 2015 recommend the use of T3 as an augmentation agent and The CANMAT 2023 update on Clinical Guidelines for Management of Major Depressive Disorder in Adults also recommend T3 as an augmentation agent where monotherapy has failed (56, 57). The TMAP guidelines recommend T3 as a stage 1 adjunct (54).

The THESIS survey also showed large inter-regional differences in the views on prescribing TH in euthyroid patients with TRD, as high as 8.6% in Eastern Europe and as low as 4.2% and 5% in Western Europe and Northern Europe respectively (**Table 2**). The overall rate in Southern Europe was 5.5% however rates in Italy (1.7%) and Spain (5.3%) were more in keeping with Western and Northern European rates while rates in Greece (8.8%) and Serbia (22.2%) were consistent with prescribing patterns observed in Eastern Europe. We did not have antidepressant prescribing data available for all countries who participated in the THESIS survey, however a statistically significant relationship between these rates and the views of respondents in our survey was not observed. A potential cause for this may have been the increased medication adherence in countries with higher GNI, despite the rate of prescribing having no correlation to countries’ GNI (73). A previous study published in 2010 reported the highest percentage of patients taking prescribed anti-depressants regularly was Sweden. The lowest rates of adherence were reported in Italy, Slovak Republic and Czech Republic (73). Medication cost may have been an issue in some of the countries surveyed. The linear regression (**Figure 2**) showed a statistically significant relationship with decreasing rates of TH prescribing in biochemically euthyroid patients with TRD with increasing GNI. This large variation of GNI in countries in Southern Europe may explain the differing views of respondents in this region. It is possible that specialists in low GNI countries may have a need to use alternative agents for the treatment of TRD more than their Western or Northern European peers due to medication costs. OECD data show that each of Spain, Germany and Italy spent over 19 times more than each of Romania, Hungary or the Slovak Republic in 2022 (74).

### Relevance, strength and limitations of the THESIS study

The THESIS survey is the largest survey conducted on the use of TH with respect to the number of participating countries and also number of valid responses. Given the volume, aggregate responses are likely to represent practices throughout Europe (22, 23). The response rate is similar to previous comparable surveys (75, 76). Therefore, the data collected by the THESIS survey is most likely representative of the views of European specialists on TH use. Data have previously been published on the use of TH in Australia and Latin America and are now being expanded to other continents (77, 78).

Limitations in this study include definitions in the question posed in the survey. The question posed to respondents was whether TH was indicated in euthyroid patients with TRD. It is possible that this may have been interpreted differently by respondents, and some may not have included T3 when considering the question, given the lower evidence for T4 as an adjunct for TRD. Potentially different responses would have been received, had T3 been specified. Another limitation of this study is the varying definition of TRD. While it is commonly described as previously in this paper, there are varying definitions which may have impacted the responses in our survey. It is also possible that this definition may change in different regions surveyed. We did not seek the volume of patients with depression or TRD, which individual respondents encounter in clinical practice that may have given better insight into prescribing practices. Primary care physicians were also not surveyed, who are typically the first to treat both patients with depression and hypothyroidism (79). Limitations also include selection bias inherent in volunteering to participate in surveys.

## Conclusions

The majority of respondents to the THESIS survey (93.9%) do not believe that TH is indicated in biochemically euthyroid patients with TRD. Of the 348 respondents (6.1%) who do believe that it is indicated, this view was associated with (i) respondent characteristics: male, higher volume of hypothyroid patients, private practice and not a member of an international professional organization and (ii) national characteristics: Eastern European, low GNI. Prescribers should be aware that the prescription of TH in biochemically euthyroid patients with TRD may lead to iatrogenic and the misuse of T3 should be addressed, not only in hypothyroidism but also in all non-hypothyroid conditions (80, 81). The literature does not support the use of T4 in the treatment of TRD and it is not currently recommended in any guidelines. Due to limited evidence, the use of T3 in TRD is not currently supported by professional endocrinology associations however more research is needed in this area. Our findings raise questions regarding the use of TH in TRD across Europe and highlight the need for further studies investigating TH as an augmentation agent in TRD, as well as a consensus position from both endocrinology and psychiatry bodies regarding the use of TH in TRD.

## Data Availability

The datasets presented in this article are not readily available because Data is the property of the THESIS sub-committee. Requests to access the datasets should be directed to ludgates@tcd.ie.
